# Reliability and validity evaluation of the chinese version of the ethical sensitivity questionnaire for nursing students

**DOI:** 10.1186/s12912-021-00768-z

**Published:** 2021-12-06

**Authors:** Haitao Yu, Tong Tong, Ye Gao, Hui Zhang, Huijuan Tong, Chunguang Liang

**Affiliations:** 1 School of Nursing, Jinzhou Medical University, No 40, Section 3, Songpo Road, 121001 Jinzhou, China; 2 Liaoning Vocational University of Technology, Jinzhou, China; 3 School of Nursing, Shenyang Medical College School, Shenyang, China

**Keywords:** Ethical sensitivity, Nursing students, Validity, Reliability

## Abstract

**Background:**

Advances in technology and the expansion of nursing roles have led to complex ethical issues in nursing. Nursing students are the future clinical nursing workers and practitioners. The ethical sensitivity of nursing students is very important to the professional development of nursing students, which can strengthen the ethical cognition of nursing students, improve the ethical decision-making ability of nursing students, and is beneficial to the development of nursing students in the process of clinical practice and nursing education. However, there are no instruments to evaluate the ethical sensitivity of nursing students in China. Therefore, the purpose of this study is to evaluate the validity and reliability of the Chinese version of the Ethical Sensitivity Questionnaire for Nursing Students (ESQ-NS).

**Methods:**

After obtaining the authorization of the author of the original scale, the study used the Brislin back-translation method for translation. An exploratory factor analysis (EFA) and a confirmatory factor analysis (CFA) were performed to examine the underlying factor structure of the translated questionnaire. The Cronbach alpha coefficient, the test-retest reliability, and the corrected item-total correlation were calculated to verify the internal consistency of the scale.

**Results:**

The Chinese version of ESQ-NS retained 13 items. Exploratory factor analysis (EFA) extracts four common factors, and the cumulative variance contribution rate is 62.479%. The CFA reached the adaptive standard, and the discriminant validity of the scale was good. The Cronbach alpha coefficient of this scale was 0.821, and 4 dimensions were between 0.708 and 0.738. The results of the test-retest showed that Pearson’s correlation coefficient of the overall ESQ-NS was 0.814. Corrected item-total correlation ranged from 0.337 to 0.542.

**Conclusions:**

The Chinese version of the ESQ-NS has good reliability and validity, which can be used to evaluate the level of ethical sensitivity of nursing students in China.

## Introduction

With the rapid development of high and new medical technology and the current social transformation period, there have been some new changes in the nurse-patient relationship [1]. The work of nurses is no longer a simple operation of basic skills such as injection and medicine dispensing, but we need to constantly explore the new changes in the new era of nursing ethics, so as to guide the cultivation of the professional ethics of nurses, nursing professional elements and ethical requirements internalized into nurse behavior habits [2–4]. Therefore, it is particularly important to pay attention to the cultivation of ethical sensitivity. Ethical sensitivity is an important part of nursing practice. No matter the in-role behavior required by the position or the spontaneous out-of-role behavior, they are all in the ethical context of communicating and dealing with patients [5]. The ability to identify ethical problems is a lifelong learning process for nursing staff. Ethical sensitivity has been proved to be related to understanding ethical dilemma and dealing with ethical conflict, cultivating clinical ethical decision-making ability, forming ethical professional accomplishment and practicing ethical behavior norms [6–8].

Ethical sensitivity is a positive attribute in theory, and awareness of the existence of ethical issues is a prerequisite for decision-making and taking actions [9]. Improving the ethical sensitivity of nursing students can strengthen the ethical cognition of nursing students and enhance the practice ability of clinical decision-making [10]. In the face of the new pattern of health development, nursing students must keep up with the trend and constantly improve their own ethical sensitivity. High ethical sensitivity is conducive to improving the ability of combining nursing students’ professional level with nursing ethics and moral quality. Lack of ethical sensitivity will lead to the loss of moral responsibility and negative health consequences.

Ethical sensitivity is an evolving concept. Rest [11] defined ethical sensitivity as the ability to make ethical decisions without obvious ethical conflicts and to evaluate the reactions and feelings of others and to recognize their relative importance to the patient and the potential course of action to take. Lützén and colleagues [12] pointed out that ethical sensitivity is the ability to show contextual and intuitive understanding of patients’ vulnerable situations, to recognize ethical conflicts and to have insight into the ethical consequences of their decisions.

Previous studies have shown that the Moral Sensitivity Questionnaire (MSQ) developed by Lützén and colleagues [12] was often used to measure ethical sensitivity among nurses in Japan [13] and Korea [14]. However, China lacks such instruments. Although some studies have also used the Moral Sensitivity Questionnaire-Revised Version into Chinese (MSQ-R-CV) translated by Huang et al. in 2015 [15], which is mainly used by nurses or professionals. Currently, there is a lack of effective tools to assess the level of ethical sensitivity of nursing students. This must be urgently addressed so that the education and training sector can be kept informed.

Taeko Muramatsu and colleagues [16] defined the ethical sensitivity of nursing students as the ability to identify ethical issues in nursing practice and developed a validated instrument called the Ethical Sensitivity Questionnaire for Nursing Students (ESQ-NS). Altogether, 13 items for nursing students were identified and classified into there domains: respect for individuals, distributive justice, and maintaining patients’ confidentiality. Studies [17–19] have shown that ethical sensitivity can be improved through training and education. The measuring instrument of ethical sensitivity can help nursing students understand their sensitivity level more clearly, which is conducive to making up for their shortcomings. It can promote nursing students in the future nursing practice can be sensitive to find the patient’s problems, understand the patient’s situation, as far as possible to avoid the occurrence of nurse-patient conflict. In a complex and challenging health care environment, this is critical for the future development of nursing students. The purpose of this study was to translate ESQ-NS into Chinese and to describe the reliability and validity of the Chinese version of ESQ-NS among nursing students.

## Methods

### Study design and participants

A cross-sectional survey was conducted from August to November 2020 in Liaoning Province, China. Participants included first-year to fourth-year nursing students from Shenyang (China Medical University, Liaoning University of Traditional Chinese Medicine, Shenyang Medical College) and Jinzhou (Jinzhou Medical University). The research procedures complied with the ethical standards of the institutional research committee, as well as the 1964 Helsinki declaration and its later amendments. All participants were given detailed information about the purpose and methodology of the study. We will ensure the information security of everyone, and have signed informed consent before preparing the questionnaire.

### Instruments

#### Ethical Sensitivity Questionnaire for Nursing Students (ESQ-NS)

Ethical Sensitivity Questionnaire for Nursing Students developed by Taeko Muramatsu and colleagues [16] consists of 13 items, covering three dimensions: respect for individuals (8 items), distributive justice (3 items), and maintaining patients’ confidentiality ( 2 items). The respondent is asked to rate each item on a 4-point Likert scale ranging from 1 for “strongly disagree” to 4 for “strongly agree”. The ESQ-NS score ranges from 13 to 52, with higher score indicating higher ethical sensitivity. The Cronbach alpha coefficient of this scale was 0.821.

#### The Chinese Moral Sensitivity Questionaire-Revised Version (MSQ-R-CV)

The Chinese Moral Sensitivity Questionaire-Revised Version (MSQ-R-CV) was translated into Chinese by Huang [15] after strict cultural adjustment. The scale consists of 9 items and contains 2 dimensions: moral responsibility and strength (5 items), sense of moral burden (4 items). The respondent is asked to rate each item on a 6-point Likert scale ranging from 1 for “strongly disagree” to 6 for “strongly agree”. The MSQ-R-CV score ranges from 9 to 54, with higher score indicating higher moral sensitivity. The Cronbach alpha coefficient of the MSQ-R-CV was 0.82. The MSQ-R-CV was used to measure concurrent validity.

#### Demographic characteristic

Demographic measures included age, sex, school year, clinical experience, ethical education, and nursing professional attitude.

### Procedures

#### Translation procedure

The original questionnaire was translated into Chinese by two nursing experts respectively, and then back-translated into English by two English experts according to the Brislin translation method [20]. The original questionnaire, the first draft of the Chinese version and the translated English questionnaire were discussed, compared and revised by a psychology expert and a nursing expert who is familiar with Chinese and Western cultural, so as to make the contents of the questionnaire more consistent with Chinese cultural habits. Finally, we randomly elected 10 students to assess the revised scale. Based on their feedback, the scale was revised and improved.

#### Data collection procedure

The students were told that the purpose and significance of the study. Before the beginning of the offline survey, the investigators were uniformly trained, and the investigators explained each item before the students filled in the questionnaire, so as to ensure that each student had no doubt about the contents of the questionnaire. After the recall, the questionnaires were numbered one by one, and the double-entry principle was implemented to ensure the accuracy of the data. A total of 1465 nursing students participated in the study, and 1446 questionnaires (98.70%) were completed for analysis.

### Statistical analysis

Data analysis used SPSS 25.0 and AMOS 23.0 (IBM Corporation). Continuous data were expressed as mean (SD) and categorical data as percentages. Calculated the skewness and kurtosis of each item. When the value is between -2 and +2, the data is considered to be normally distributed [21]. Content validity, structure validity, discriminant validity, concurrent validity, internal consistency, retest reliability and the corrected item-total correlation of the ESQ-NS scale were measured in our study.

### Content validity

Content validity refers to the appropriateness and consistency between the actual measured content and the content to be measured [22]. Seven experts were invited to evaluate the content validity of the Chinese version of ESQ-NS. Each item is scored on a four-point scale from 1 to 4. The scores indicated no relevance, low relevance, strong relevance and very strong relevance. Content validity was calculated according to experts scores, including item-level content validity index (I-CVI) and average scale-level content validity index (S-CVI/Ave).

### Structure validity

Structure validity is an indicator to measure whether the tool scores fully reflects the dimension structure [23]. To explore the underlying factor structure of the translated questionnaire, an exploratory factor analysis (EFA) and a confirmatory factor analysis (CFA) were performed. The sample of 1446 cases was randomly divided into 2 groups, one (*n*=747) for EFA and the other (*n*=699) for CFA. EFA was used to determine the structural validity of the Chinese version of the scale. Calculated value of the Bartlett test [24] of sphericity was significant (*P*<0.05) and the Kaiser-Meyer-Olkin (KMO) [25] was >0.60, indicating that it was suitable for factor analysis. Principal component analysis and varimax rotation method were used to extract common factors with eigenvalues greater than 1. Based on the factor division obtained by exploratory factor analysis, AMOS (IBM Corporation) is used to construct the CFA model, analyzing the fit of models and its parameter estimates.

### Discriminant validity

The purpose of discriminant validity is to judge whether there is a good discriminant degree between different latent variables [26]. The discriminant validity of the Chinese version of ESO-NS was analyzed by using a 2-tailed independent samples t test. The total score of the ESQ-NS scale was ranked from low to high; the bottom 27% of the scores were grouped into the low-score group, and the top 27% of the scores were grouped into the high-score group.

### Concurrent validity

Concurrent validity is a kind of criterion validity, which refers to the use of recognized valid scales as standards to test the correlation between the new scale and the standard scale [27]. Pearson correlation coefficient between ESQ-NS and Chinese version of MSQ-R-CV was used to analyze the concurrent validity. It is generally believed that the concurrent validity is between 0.4 and 0.8 [28].

### Reliability analysis

The internal consistency of the scale was tested by calculating the Cronbach alpha coefficient and the corrected item-total correlation. The time stability of the scale was evaluated by retest reliability. After an interval of 14 days, 30 nursing students were randomly selected to complete the scale. In this study, the retest interval of the scale’s retest reliability was two weeks. The reason was based on the principle that nursing students basically forget the survey content and are not affected by other environmental factors [29]. It was generally accepted that Cronbach alpha coefficient and retest reliability greater than 0.70 were acceptable [30]. Using 0.3 as the inclusion criterion to judge the corrected item-total correlation [31].

## Results

### Descriptive statistics

The study included 1446 nursing students. The mean age of the participants was 21.3±1.9 years. Nursing students who were female (90.9%), 2nd grade (37.1%) count the most, non-clinical experience(52.8%), and had ethics education(82.5%), liked the nursing profession(43.0%). Other characteristics are presented in Table 1. The mean (SD) score of each item of the Chinese ESQ-NS is shown in Table 2. These data were normally distributed according to the skewness and kurtosis figures.

**Table 1:** Demographic characteristics of participants (*N* = 1446)

Characteristics	N	%
Sex		
Male	131	9.1
Female	1315	90.9
Age(mean, standard deviation)	21.3±1.9	
School Year		
1st grade	377	26.1
2nd grade	537	37.1
3rd grade	265	18.3
4th grade	267	18.5
Clinical experience		
Yes	683	47.2
No	763	52.8
Ethics education		
Yes	1193	82.5
No	253	17.5
Attitudes towards the nursing profession		
Like it very much	261	18.0
Like	622	43.0
General	563	38.9

**Table 2:** Mean (SD) scores with skewness and kurtosis figures (*N*=1446)

Item	Mean(SD)	Skewness	Kurtosis
1	3.25(0.813)	- 0.128	0.666
2	3.11(0.767)	- 0.648	0.172
3	2.93(0.841)	- 0.317	- 0.645
4	2.61(0.790)	- 0.049	- 0.047
5	3.24(0.734)	- 0.937	1.067
6	2.53(0.897)	- 0.003	- 0.762
7	2.62(0.808)	- 0.163	- 0.438
8	2.33(0.985)	0.032	- 1.023
9	2.93(0.823)	- 0.554	- 0.083
10	2.91(0.768)	- 0.502	0.116
11	3.00(0.719)	- 0.502	0.334
12	2.73(1.009)	- 0.352	- 0.953
13	2.42(0.993)	0.090	- 1.036

### Validity analysis

#### Content validity

The content validity of Chinese version of ESQ-NS was evaluated by expert evaluation method [32]. The expert group consists of 7 experts, including 3 nursing ethics experts, 2 nursing education experts and 2 nursing experts who are proficient in Chinese and English. The results of content validity analysis showed that I-CVI of ESQ-NS is 0.857~11.000, and the S-CVI / Ave is 0.956.

#### Explorary factor analysis

**Fig. 1:**
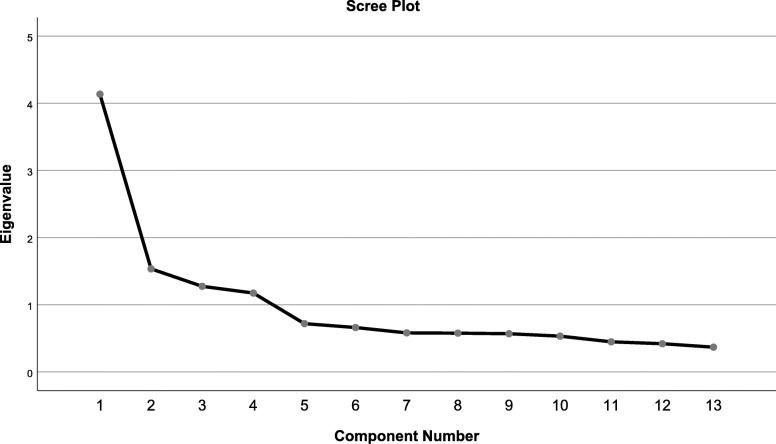
Scree plot of exploratory factor analysis for Chinese version of the ESQ-NS

The Bartlett test [24] of sphericity was significant (χ^2^_78_=2551.037; *P*<0.001), and the KMO index [25] was 0.829, greater than the minimum acceptable value of 0.6, which confirmed the feasibility of factor analysis. Principal component analysis and varimax rotation extracted four common factors and explained 62.479% of the variance. Table 3 showed the factor loading for each item. According to the previous research, a factor loading of 0.4 was selected as the cut-off point to retain the items [33]. The scree plot further confirmed the 4-factor structure, and the descending tendency became weak after the fourth point, indicating that it was appropriate to select four common factors. After varimax rotation, the four factors explained 17.339%, 16.683%, 15.685% and 12.772%, respectively. Figure 1 shows the scree plot.

**Table 3:** Exploratory factor analysis (*N* = 747)

Original structure	Factor 1		Factor 2	Factor 3
Modified structure	Factor 1	Factor 2	Factor 3	Factor 4
1. Railing is placed around a bed to prevent the patient from falling out.	0.757			
2. Although a postoperative patient has refused postural changes due to pain, postural changes are performed to prevent postoperative complications.	0.799			
3. Although a terminally ill patient has refused postural changes due to respiratory discomfort caused by moving, postural changes are performed every two hours due to the high risk of pressure ulcers.	0.620			
5. A sensor mat is placed at the bedside of a patient who had fallen once in the ward.	0.617			
4. An elderly patient who had said he/she wanted to go home was placed in a facility because he/she had no relatives who could care for them at home.		0.663		
6. You allowed a patient with dementia to stay at the nurses’ station while sitting in a wheelchair with the safety-belt fastened.		0.681		
7. A patient under your care who was of the opposite sex had refused to let you watch over him/her when he/she showered; however, you did so after persuading him/her to allow you to.		0.676		
8. To administer medication to a patient with dementia who refuses medication, it is mixed with a drink without the patient’s knowledge.		0.756		
9. A terminally ill patient wished to use the bathroom for elimination; therefore, two nurses took the patient to the bathroom and aided.			0.736	
10. A bedridden patient who had always received a bed bath pleaded to take a regular bath; therefore, three nurses assisted the patient in taking a regular bath.			0.832	
11. To accommodate the eating speed of patients with dysphagia, you provide eating assistance that involves uninterrupted supervision for at least one hour.			0.704	
12. Reporting the condition of a patient under your care to the nurse in charge in a multi-bed hospital room.				0.852
13. Reporting the details of the patient care to a clinical leader in the corridor.				0.803

#### Confirmatory factor analysis

**Fig. 2:**
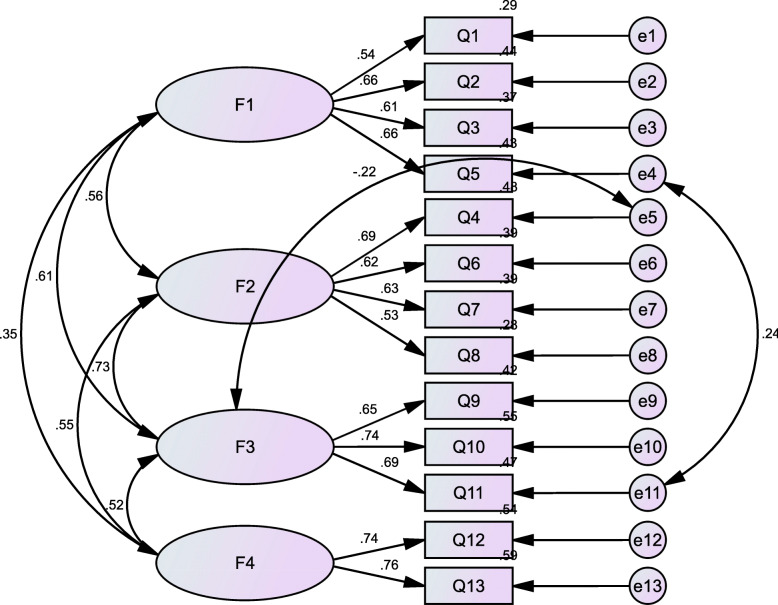
Standardized four-factor structural model of the ESQ-NS (*n*=699). F1 (Respect for individuals, four items), F2 (Reasonable care, four items), F3 (Distributive justice, three items), F4 (Maintaining patients’ confidentiality, two items).

Confirmatory factor analysis was performed on 699 nursing students using a four-factor model. The present model provides an acceptable fit to the data. Fit indexes are as follows: CMIN/DF (chi-square/degree of freedom)=2.712, CFI (comparative fit index)=0.959, GFI(goodness of fitness index)=0.966, AGFI (adjusted goodness of fit index)=0.946, PGFI (parsimonious goodness of fit index) =0.605, IFI (incremental fit index)=0.959, TLI (Tucker Lewis index)=0.944, RMSEA (root mean square error of approximation)=0.050, and RMR (root mean residual)=0.030. The results of CFA are shown in results are shown in Fig. 2.

#### Discriminant validity

In this study, extreme grouping method was used to analyze the discriminant validity. The total scores were ranked from high to low, and the critical value was 27%. The scores≥39 were classified as high group, and≤33 were classified as low group. The results showed that the scores of each item had statistical significance in both high and low areas (*P* < 0.05). This indicates that each item of ESQ-NS has a good degree of discrimination, and the discrimination ability of each item of the scale is good. The results are shown in Table 4.

**Table 4:** Score comparison between high-score and low-score groups (*N*=1446).

Item	Low-score group (*n*=404), mean (SD)	High-score group(*n*=498), mean (SD)	t test (df)	*P* value
1	2.87(0.936)	3.58(0.610)	- 13.202(664.457)	<0.001
2	2.70(0.832)	3.49(0.596)	- 16.114(708.504)	<0.001
3	2.37(0.800)	3.43(0.690)	- 21.187(799.765)	<0.001
4	2.10(0.641)	3.11(0.733)	- 21.888(894.983)	<0.001
5	2.79(0.862)	3.61(0.524)	- 16.752(635.096)	<0.001
6	1.95(0.770)	3.12(0.786)	- 22.4000(868.918)	<0.001
7	2.13(0.728)	3.09(0.735)	- 19.710(900)	<0.001
8	1.75(0.775)	2.91(0.909)	- 20.337(900)	<0.001
9	2.36(0.851)	3.39(0.656)	- 19.828(745.244)	<0.001
10	2.39(0.766)	3.35(0.642)	- 20.033(786.4491)	<0.001
11	2.50(0.751)	3.42(0.573)	- 20.197(739.981)	<0.001
12	2.02(0.928)	3.35(0.778)	- 32.023(786.733)	<0.001
13	1.77(0.802)	3.05(1.913)	- 22.094(900)	<0.001

#### Concurrent validity

The Pearson correlation between ESQ-NS total score and MSQ-R-CV total score was 0.488, indicating a good correlation. This means that the measurement tool can effectively measure the characteristics of the concept to be measured.

#### Reliability analysis

The results of reliable analysis show that ESQ-NS had ideal internal consistency. The internal consistency of alpha was 0.821, and the four dimensions of Cronbach alpha coefficient were 0.714, 0.708, 0.738 and 0.710, respectively. Table 5 shows the change of Cronbach alpha value after the exclusion of a particular item from the questionnaire. The corrected item-total correlation of the items ranged from 0.337 to 0.542, all greater than 0.3. Two weeks later, 30 nursing students were randomly selected to evaluate the reliability of the scale retest. The retest reliability was 0.814.

**Table 5:** Cronbach alpha coefficient if the item was deleted and corrected item-total correlation (*N*=1446).

Item	Cronbach alpha if the item was deleted	Corrected item-total correlation
1	0.818	0.337
2	0.812	0.425
3	0.807	0.486
4	0.806	0.508
5	0.809	0.464
6	0.807	0.490
7	0.806	0.499
8	0.814	0.413
9	0.807	0.485
10	0.806	0.508
11	0.804	0.542
12	0.811	0.444
13	0.812	0.441

## Discussion

The Chinese version of ESQ-NS scale strictly followed the procedures of translation, back-translation, cultural adjustment and pre-survey [20]. At each stage, the experts discussed and modified fully, and according to the Chinese guidelines and common expressions, the Chinese version of ESQ-NS was obtained.

The results of this study showed that the items of the Chinese version of ESQ-NS were consistent with those of the original scale, and no items were deleted. However, the factor structure of the Chinese version is slightly different from that of the original one. The original ESQ-NS had three dimensions: respect for individuals (Item 1-8), distributive justice (Item 9-11) and maintaining patients’ confidentiality (Item 12-13), with a total of 13 items. In this study, exploratory factor analysis showed that the first factor had 4 items, involving the original scale 1, 2, 3 and 5, named “respect for individuals”. Factor 2 had 4 items in total, involving the original scale 4, 6, 7 and 8. Based on expert opinions, literature review and the underlying characteristics of the items, we renamed it “reasonable care”. The dimensions of factor 3 and factor 4 were exactly the same as those of the original scale, indicating that they were easier to apply cross-culturally. An EFA determined 13 items categorized under four factors (respect for individuals, reasonable care, distributive justice, maintaining patients’ confidentiality), which explained 62.479% of the total variance. Each item had a factor loading of 0.60 or higher, which was considered ideal [34].

The researchers interpreted and labeled the emerging factor according to the core concepts of the human care theory by WATSON J [35]. The second factor (“reasonable care”) explains 16.683% of the total variance and should be considered as the main aspect for the existence of a valid assessment of ESQ-NS level. Watson’s theory points out that care is the essence of nursing, which provides theoretical core knowledge for patient care and provides a new dimension for revealing the core essence of nursing. Literature [36, 37] have revealed that in the analysis and application of human care theory, the dynamic change of human care needs in different stages plays an important role in clinical nursing practice, nursing education, nursing management and other fields. In fact, studies [38, 39] have shown that due to the lack of safe nursing knowledge and medication methods. Many patients have been admitted to professional institutions for treatment or formal care, which also suggests that nursing staff should strengthen the study of basic principles and ethical requirements of care. Nursing students are the reserve force of medical service application-oriented talents. Their willingness to care directly affects the quality of future care system professionals [40]. Therefore, we use “reasonable care” as an independent factor in evaluating ethical sensitivity tool, rather than being included among other factors.

In our study, in addition to the three basic aspects of the original version, our scale includes “reasonable care” (factor 2), which makes the domain classification of the Chinese scale more specific. Considering that the incidence of nurse-patient relationship, nurse-patient disputes, nurse-patient conflicts and other cases is increasing year by year, nursing students believe that the focus of professional nursing is to care patients, hope to take better care of patients through their own ability, and have a stronger sense of responsibility for taking care of patients [41]. This may be influenced by different medical environment, cultural background and subject education at home and abroad. Different regions have different educational modes for nursing students, so they have different understandings of problems. Further research is needed to see if the findings can be applied to other cultures.

According to the expert evaluation method, the content validity range of I-CVI of each item level was 0.857~1.000 ( I-CVI≥0.78) [22]. The value of I-CVI ranges from 0 to 1. A larger value indicates that the item is more representative and more suitable for the items in the scale. The S-CVI/Ave of ESQ-NS reached 0.956 (>0.90), indicating that the scale has good content validity [42]. The analysis shows that the Bartlett spherical test<0.01, KMO>0.6, with a value of 0.857 indicating a moderate-to-high level of adequacy of the correlation matrix, which shows that the data are suitable for factor analysis. Four common factors were obtained through CFA analysis, each index has good adaptability and within a reasonable range (CMIN/DF<3, CFI, IFI, GFI, AGFI,TLI>0.90,PGFI>0.50,RMR<0.05, RMSEA<0.08). The results prove that factor loading and interpretation variance are strong, which are consistent with EFA results, and have a good four-factor structure and model fitting index. The findings showed that the Chinese version of ESQ-NS could reflect the ethical level of nursing students and the function of nursing students in four dimensions. A significant positive correlation was found between ESQ-NS and MSQ-R-CV (*r*=0.488, *P*<0.001). The results show that the concurrent validity was well.

In the ESQ-NS, the internal consistency of the Chinese version of ESQ-NS and its 4 dimensions, as measured by Cronbach alpha coefficient, were all greater than 0.7. The internal consistency were consistent with those of the original study [16]. Corrected item-total correlation are greater than 0.3. Test-retest reliability of the whole scale was 0.814. All result show that the scale is stable and all indexes are within a reasonable range, so it can be considered as a reliable assessment tool to be applied to Chinese nursing students.

## Limitations

This study has shortcomings. The participants of this study were only college students from Liaoning Province, and female participants were significantly higher than male participants. Therefore, these findings do not represent wishes of all nursing students in China. Attention should be paid to the ratio of gender of samples, expand the coverage of samples, and further evaluate the adaptability of the scale of ethical sensitivity of nursing students in the whole country.

## Conclusions

Ethical sensitivity is a key factor in the ethical education and nursing practice of nursing students. An increasing number of health care researchers pay attention to it. Effective and feasible assessment tools for ethical sensitivity are essential for nursing educators and nursing students. The Chinese version of the instrument, which supports a four-factor structure, proved to be reliable. In future research, we believe that it is necessary to use the ESQ-NS at the end of every academic year’s theoretical and practical courses to assess ethical sensitivity of nursing students, so as to provide an empirical basis for effectively improving nursing students’ ethical decision-making ability and preventing ethical dilemmas.

## Data Availability

The datasets used and/or analysed during the current study are available from the corresponding author on reasonable request.
